# Proteomic Analysis of Albumins and Globulins from Wheat Variety Chinese Spring and Its Fine Deletion Line 3BS-8

**DOI:** 10.3390/ijms131013398

**Published:** 2012-10-18

**Authors:** Chao-Ying Ma, Li-Yan Gao, Ning Li, Xiao-Hui Li, Wu-Jun Ma, Rudi Appels, Yue-Ming Yan

**Affiliations:** 1 College of Life Science, Capital Normal University, Beijing 100048, China; E-Mails: machao3721@sina.com (C.-Y.M.); gaoliyan2001@163.com (L.-Y.G.); liningxinnian@126.com (N.L.); lixiaohui1978@163.com (X.-H.L.); 2 State Agriculture Biotechnology Centre, Murdoch University, Western Australian Department of Agriculture and Food, Perth, WA 6150, Australia; E-Mails: proteomics@gmail.com (W.-J.M.); rudiappels@gmail.com (R.A.)

**Keywords:** Chinese spring, 3BS-8, albumins, globulins, proteome

## Abstract

The relationship between chromosome deletion in wheat and protein expression were investigated using Chinese Spring and fine deletion line 3BS-8. Through 2-DE (2-D electrophoresis) analysis, no differentially expressed proteins (DEPs) were found in leaf samples; however, 47 DEPs showed at least two-fold abundance variation (*p* < 0.05) in matured wheat grains and 21 spots were identified by tandem MALDI-TOF/TOF-MS. Among the identified spots, four were cultivar-specific, including three (spots B15, B16, and B21) in Chinese Spring and one in 3BS-8 (spot B10). Among variety-different DEPs between Chinese Spring and 3BS-8, most spots showed a higher express profile in CS; only four spots showed up-regulated expression tendency in 3BS-8. An interesting observation was that more than half of the identified protein spots were involved in storage proteins, of which 11 spots were identified as globulins. According to these results, we can presume that the encoded genes of protein spots B15, B16, and B21 were located on the chromosome segment deleted in 3BS-8.

## 1. Introduction

As a worldwide food crop, bread wheat is a hexaploid species with three related diploid genomes denoted as A, B and D, and is composed of seven pairs of chromosomes in each genome. In the last few decades, extensive research has been conducted for the improvement of wheat yield and grain quality, mainly at the gene level [1]. However, wheat genomics and its application to crop improvement are lagging behind those of most other important crops. For example, map based cloning in wheat is still difficult due to its large genome size, high repetitive sequence content, polyploidy nature, genome complexity and lack of the reference sequence [2].

The polyploidy nature of the wheat genome provides considerable genetic buffering that allows aneuploids and deletions to remain viable and fertile, and the chromosome deletions are stable and transmitted to the offspring without further structural changes [3]. Sears [4] developed a series of ditelocentric lines in which each line has a chromosome arm deletion for a particular chromosome. He also developed a series of nullisomic-tetrasomic lines in which each line is missing one entire chromosome for a particular genome and has two extra chromosomes of one of its homologous chromosomes. Hundreds of disomic deletion lines have been used to localize genes and molecular markers to specific regions of the chromosome [5–8]. For example, β-amylase (β-Amy-A2) in common wheat was mapped cytologically on the long arm of chromosome 5A by using four 5Aq deletion lines [9]. In proteomics researches, the deletion lines are also widely used. Francs and Thiellement [10] found 185 protein spots variable between the euploid and 26 ditelosomic lines of Chinese Spring and located 35 structural genes on 17 chromosome arms. Payne *et al.* [11] studied the wheat endosperm proteins and determined the chromosome and chromosome-arm location of most components using several ditelosomic lines of Chinese Spring. Islam *et al.* [12] reported the changes in protein composition of wheat endosperm proteome between the euploid and the 39 ditelocentric lines. They identified some proteins and located the structural genes controlling these spots in the chromosome arms. In 2003, Islam *et al.* [13] investigated the changes in protein composition of wheat seed proteome to explore the relationship between fine chromosome deletion within 1B and protein expression in common wheat.

Chromosome (3B) of common wheat is also known to be the largest wheat chromosome with a size of 995 megabases [2]. In 2008, the bacterial artificial chromosome (BAC)–based integrated physical map of chromosome 3B had been published [2]. Although there has been a lot of research on gene locations for different chromosomes fragments [10–14], the information does not necessarily match quantitatively or qualitatively at the protein level. Therefore, proteomic information of chromosome 3B is still lacking. In the present work, we performed for the first time a proteomic analysis using 3BS-8, one of the fine deletion lines of wheat chromosome 3B, to identify the specific location of genes controlling grain albumins and globulins. Our results provide valuable information for further studies on wheat genomics and proteomics as well as genetics and breeding.

## 2. Results and Discussion

### 2.1. Molecular Identification of 3B Fine Deletion Lines by PCR

**Figure 1:**
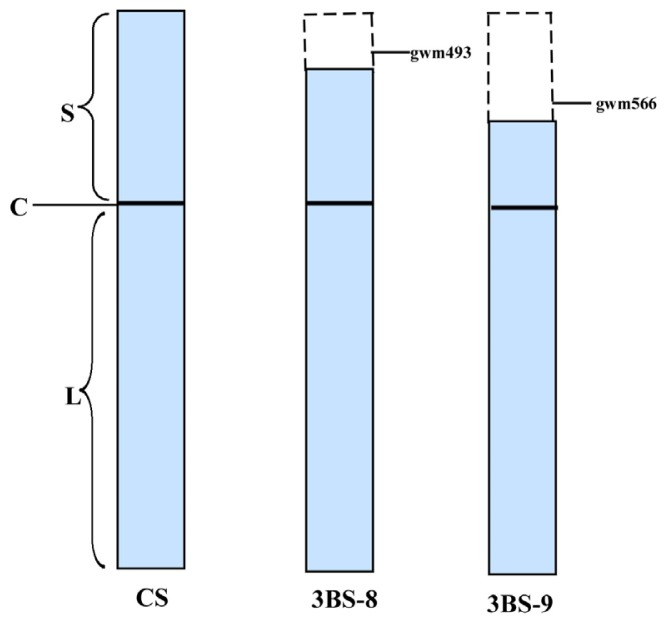
Diagrams of 3B chromosome of Chinese Spring (CS) and its fine deletion lines. The dashed boxes indicated the deleted chromosome segments gwm493 and gwm566. S–short arm; L–long arm; C–centromere.

By C-banding, Endo and Gill [6] detected 23 deletion lines on chromosome 3B with different breakage points from telomere to centromere. Among these, 3BS-8 is a fine deletion line derived from Chinese Spring (CS), which is characterized by a terminal deletion on the short arm of chromosome 3B. The locations of deleted chromosome segments in two 3B fine deletion lines were shown in Figure 1. A shorter and longer segment in the short arm of 3B chromosome was lost in 3BS-8 and 3BS-9, respectively. Microsatellite markers Xgwm493 and Xgwm566 are located distal to the breakpoint 3BS-8 and between 3BS-8 and 3BS-9, respectively [15].

**Figure 2:**
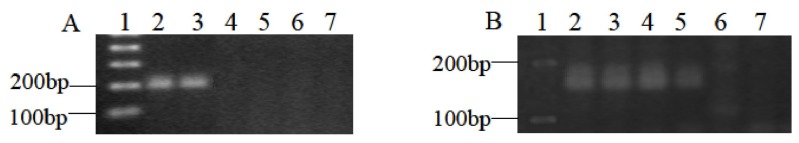
Amplifications of Chinese Spring and fine deletion lines. A and B showed the amplification products of gwm493 and gwm566, respectively. (1: DNA size marker; 2 & 3: Chinese Spring; 4 & 5: 3BS-8; 6 & 7: 3BS-9).

In order to confirm the authenticity of the fine deletion line 3BS-8, microsatellite markers Xgwm493 and Xgwm566 were tested by using 3BS-9 as control (Figure 2). The results showed that Xgwm493 had a clear amplification of 200 bp in CS, but no amplified products in either 3BS-8 or 3BS-9. When primer gwm566 was used, a product of about 180 bp was amplified in both CS and 3BS-8, but no products in BS-9. These PCR results confirmed that the purity and authenticity of 3BS-8 as shown in Figure 1.

### 2.2. Morphological Characters of CS and Fine Deletion Line 3BS-8

**Figure 3:**
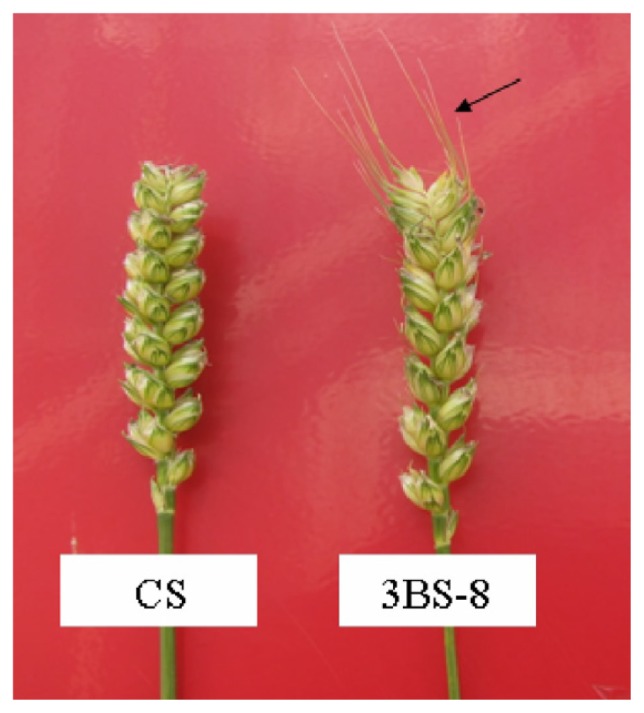
Spikes of euploid Chinese Spring and deletion line 3BS-8 (awns by arrow).

Morphological observation during plant growth and development showed that there were no significant differences in CS and 3BS-8 except for an awn phonotype. The fine deletion line of 3BS-8 had a few awns at whereas the euploid CS had awnless spikes (Figure 3). Since both varieties were grown under the same environment, the deletion fragment in chromosome 3B in 3BS-8 could be responsive for the morphological differences.

Awns may play roles in the yield in wheat, especially under drought conditions and only a few genes were involved in the genetic control of this trait [16]. Previous research showed that five major genes (*B1*, *B2*, *B3*, *A* and *Hd*), alone or in combination, lead to the production of awn phenotype [17]. Three dominant awn-inhibitors *B1*, *B2* and *Hd* were located on the long arm of chromosome 5A, 6B and the short arm of chromosome 4A, respectively [4,15,18]. Wheat genotypes containing one of these inhibitors (such as in Chinese Spring) are awnless [16]. In addition, some of the awn controlling genes are located on these corresponding chromosomes of common wheat. To date, no direct relations have been established between chromosome 3B and awn development. We speculate that the deletion fragment of chromosome 3B in 3BS-8 may contain regulatory factors that can activate the CS *B2* gene expression.

### 2.3. Leaf Proteome Analysis of Chinese Spring and 3BS-8

**Figure 4:**
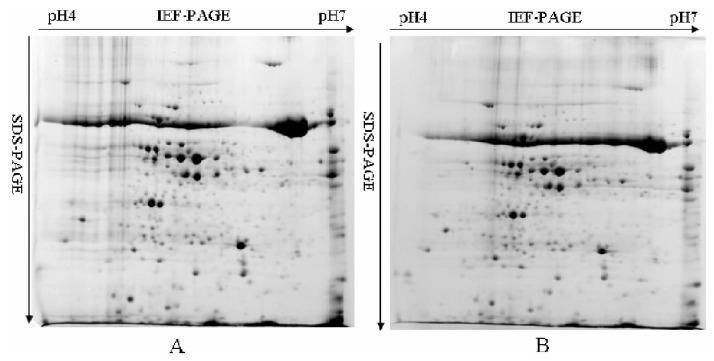
2-DE patterns of leaf albumins and globulins from Chinese Spring (**A**) and 3BS-8 (**B**).

A comparative proteomic analysis of leaf albumins and globulins from CS and 3BS-8 was carried out by quantitative 2-DE (Figure 4). In total, more than 200 protein spots were reproducibly detected on CBB stained gels, but no significant differences were found. This indicated that the lost segment of chromosome 3B in 3BS-8 has no or little effect on the expression of leaf proteins. In general, leaf proteins play important roles in the growth and development of wheat plants, and most of them belong to metabolic proteins controlled by housekeeping genes. Because the hexaploid wheat genome is highly buffered due to its polyploidy [3], and also because the loss of chromosome in 3BS-8 is a very small terminal fragments, the roles played by gene products on the deleted chromosome segment are likely to be compensated by those from other homeologous chromosomes, and consequently the growth and development of wheat plants can be maintained.

### 2.4. Proteome Analysis of Albumins and Globulins from Mature Seeds of CS and 3BS-8

**Figure 5:**
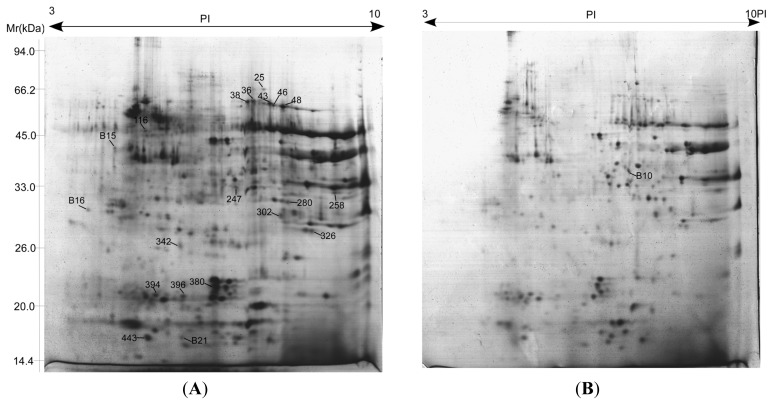
2-DE patterns of albumins and globins from mature seeds of CS (**A**) and 3BS-8 (**B**).

Albumins and globulins from mature seeds of CS and 3BS-8 were extracted and isolated by 2-DE (Figure 5). Among more than 400 spots detected in the gels, 47 showed at least two-fold abundance variation (*p* < 0.05), and 21 were identified by MALDI-TOF-MS/MS as shown in Figure 5 and Table 1. Detailed information of matched peptides was listed in Table 2. Although a similarity of protein expression profiles were observed in both lines, significant differences were also present between them. Comparative proteomic analysis demonstrated that four protein spots showed to be genotype-specific among the 21 identified differentially expressed proteins (DEPs), including triticin (spot B15), one predicted protein with unknown function (spot B16), and 0.19 dimeric alpha-amylase inhibitor (spot B21) that only appeared in Chinese Spring and one (spot B10—glyceraldehyde-3-phosphate dehydrogenase) in 3BS-8 only. According to these results, the coding genes of protein spots B15, B16 and B21 were located on the deleted chromosome segment of 3BS-8. For the other 17 DEPs between Chinese Spring and 3BS-8, 13 showed a higher express in CS, only four protein spots (spot 25-HMW glutenin subunit D^t^y10, 280-Avenin-like b1, 302-globulin 1 and 394-globulin 3) showed up-regulated expression in 3BS-8. Considering the relatively higher expression of most DEPs in CS and wheat being an allopolyploidy species, the expression of metabolic DEPs might be controlled by multiple genes that are located across the homeologous chromosomal regions. This indicates that a small chromosome fragment deletion will most likely only lead to expression decreasing of most DEPs. This explains why only a few DEPs can be located on the deleted segment in 3BS-8.

**Figure 6:**
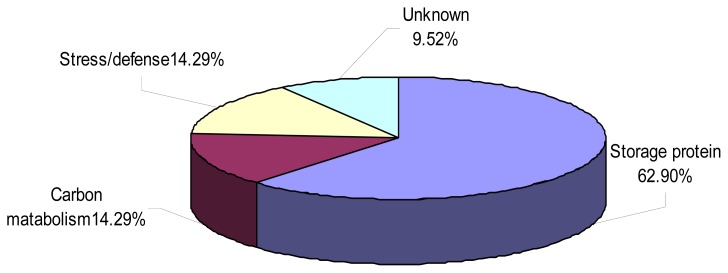
Functional distribution of 21 differentially expressed seed proteins between CS and 3BS-8.

As shown in Figure 6, the identified proteins were classified into four groups according to their functions, *viz.* storage proteins (62.9%), carbon metabolism (14.29%), detoxification, defense (14.29%) and unknown proteins including hypothetical or putative proteins with unknown functions (9.52%). An interesting observation was that 11 protein spots were identified as globulins, including three isoforms of one globulin which had seven spots (36, 38, 43, 46, 48, 394 and 396) located at different positions on the same gel. These isoforms might represent post-translationally modified forms of the same protein, such as phosphorylated and glycosylated forms [19,20]. Wheat seed proteins can induce a number of immune-mediated diseases such as Baker’s asthma in predisposed individuals [21]. Globulin-3, the *Triticum aestivum* (wheat) storage protein WP5212, was identified as the first candidate wheat protein associated with the development of type 1 diabetes (T1D) [22]. Gomez *et al.* [23] isolated and partially characterized a group of endosperm globulins in the short arms of chromosomes 1A, 1B and 1 D. Three unique wheat globulin genes, *Glo-3A*, *Glo3-B* and *Glo-3C*, were identified by screening a *Triticum aestivum* BAC genomic library [24]. Because the deletion chromosomes are stable and transmitted to the offspring [3], the specific chromosomal locations of genes identified in our study can be used as protein markers in genetics and breeding studies. The deletion line 3BS-8 may also be used as useful genetic stock for study wheat related diabetes-resistant mechanisms since the fine part deletion of chromosome 3B significantly affects the expressions of globulins related to diabetes.

## 3. Experimental Section

### 3.1. Plant Materials

Common wheat (*Triticum aestivum* L.) *cv.* Chinese Spring (CS) and two deletion lines (3BS-8 and 3BS-9), were used in this study. Both lines were grown in a climate controlled glasshouse with the maximum daytime temperature of 24 °C and minimum night time temperature of 15 °C during growth until maturity. Samples used for 2-DE analysis were collected from three replicates and stored at −70 °C prior to analysis. Half leaves from the first to the tenth leaf of the plant were combined and frozen at liquid nitrogen immediately after collecting for extraction of proteins. Mature grain samples were collected from each middle spike of two lines.

### 3.2. DNA Extraction and PCR Amplification

DNA sample was extracted from the wheat leaves as the following procedures: Fresh leaves of one to two centimeters in length were vortexed for 20 min with 300 μL DNA extration buffer I, then centrifuged at 13,000 rpm for 10 min at 4 °C. After adding 10 μL DNA extraction buffer II into 90 μL supernatant, the mixture was water bathed at 65 °C for 1 h, then centrifuged at 13,000 rpm for 2 min. Gently mixing 50 μL supernatant and 100 μL ethanal, then centrifuge the mixture at 13,000 rpm for 5 min. The pellet was dried at room temperature over night, then resolved in 100 μL 0.1 × TE buffer for another 12 h.

According chromosome locations (Figure 1), specific primers were selected and their sequences are listed in Table 3.

PCR amplification was performed in a total volume of 10 μL containing gDNA 50 ng, 3.0 μL Cresol Red, 0.25 μL MgCl_2_, 1 μL 10 × GC buffer I, 0.5 μM of each primer, 0.4 μL dNTP, 0.08 μL Taq polymerase (TaKaRa) and 2.52 μL ddH2O. The reaction was carried out according to the following procedure: For gwm493: pre-denaturation at 94 °C for 3 min, 35 cycles of 94 °C for 30 s, 60 °C for 30 s, and 72 °C for 1 min, then followed by a final cycle of extension at 72 °C for 5 min; For gwm566: pre-denaturation at 94 °C for 3 min, 8 cycles of 94 °C for 30 s, 55 °C for 30 s, 72 °C for 1 min, the annealing temperature was dropped by 1 °C every cycle; then another 25 cycles of 94 °C for 30 s, 47 °C for 30 s, and 72 °C for 1 min, then followed by a final cycle of extension at 72 °C for 5 min.

### 3.3. Protein Preparation, 2-DE and Image Analysis

Albumins and globins were extracted from 500 mg mature grain sample according to Gao *et al.* [25]. As for the extraction of leaf proteins, 400 mg sample was ground to a fine powder in liquid nitrogen using a mortar and pestle. After extracting with 3 mL extraction buffer (5 M urea, 2 M thiourea, 2% SDS, 2% Triton-114, 2 gμ/μL DTT), the suspension was incubated at 4 °C for 2 h with intermittent mixing and then centrifuged at 13, 000 rpm for 15 min at 4 °C. Three fold volumes of chilled (−20 °C) acetone were added to the supernatant and stored at −20°C overnight to precipitate the proteins, followed by centrifuging for 15 min at 13,000 rpm at 4 °C. The resultant precipitate was resuspended by 90% ethanol, kept for 5 min at room temperature, then centrifuged at 8000 rpm at 4 °C for 10 min. The resultant precipitate was washed with 1 mL ethanol for two times and with chilled (−20 °C) acetone containing 0.07% β-mercaptoethanol (ME) for three times, and was centrifuged at 8000 rpm at 4 °C for 5 min between rinses. The fluid was removed and the pellet was completely dried in a SpeedVac, then lysised with 300 μL buffer (7M urea, 2M thiourea, 4% CHAPS (3-[(3-Cholanidopropyl) dimethylammonio]-1-propanesulfonate) for 4 h. The protein samples were then centrifuged at 13,000 rpm for 10 min and the protein concentrations were determined using the Bio-Rad protein assay kit (Bio-Rad, Piscataway, NJ, USA) with bovine serum albumin as a calibration standard. Two-DE was performed according to Ge *et al.* [26]: 600 μg of protein was loaded onto analytical and preparative gels. All samples were run in triplicate to obtain statistically reliable results.

After electrophoresis, proteins were visualized by colloidal Coomassie Brilliant Blue (CBB) staining according to [26]. The 2-DE images were scanned by GS-800™ Calibrated Densitometer (BIO-RAD) and statistical analysis was performed by the ImageMaster 2D Platinum software (GE Healthcare) [27]. Determined by Student’s t test (abundance variation at least twofold, *p* < 0.05), protein spots of interest on the gels were selected for further analysis from those that showed statistically significant changes between samples.

### 3.4. Protein Identification through Mass Spectrometry

According to [26], protein spots with differential expression patterns were manually excised from gels, and then analyzed with a MALDI-TOF mass spectrometer (SM, Shimadzu Biotech, Kyoto, Japan). Unidentified spots were further analyzed using a 4800 Plus MALDI TOF/TOF™ Analyzer (Applied Biosystems, USA). All MS and MS/MS spectra were searched in the NCBI database *Viridiplantae* (900091) and *Triticum* (16682) MASCOT program (using GPS Explorer™ software version 2.0) (Applied Biosystems). The peptide tolerance was 100 ppm and fragment mass tolerance was 0.4 Da. One missed cleavage was allowed, and carbamidomethyl (Cys) and oxidation (Met) were specified as variable modifications. MASCOT scores more than 65 (*p* < 0. 05) were accepted.

## 4. Conclusions

Morphology observation demonstrated that the loss of very small terminal fragments on chromosome 3B of Chinese Spring affects the awn phenotype. Using Chinese Spring and fine deletion line 3BS-8, the relationship between chromosome deletion and protein expression was investigated in the present work. Two-DE analysis discovered 21 DEPs between the two genotypes, but only three proteins (triticin, one predicted protein with unknown function and 0.19 dimeric alpha-amylase inhibitor) can be assigned to the short arm of chromosome 3B. The deletion fragment of chromosome 3B can significantly affect the expression of seed globulins. The functional proteins on the deleted chromosome segment could be compensated by those from other chromosomes due to highly buffered wheat genome and polyploidy.

## Figures and Tables

**Table 1:** Proteins differentially expressed in the mature seeds of CS and 3BS-8.

Spot ID	Accession no.(gi)	Protein name	Species	*p*-value	Protein score a	Protein score CI%	Total ion score	Total ion score CI%	Number of matching peptides	Sequence coverage %	TpI/MW (kDa) b	EpI/MW (kDa) c
		**Storage proteins**										
36	215398470	globulin 3	*T. aestivum*	0.002	375	100	258	100	19	35.8	7.78/66.65	7.39/60.93
38	215398470	globulin 3	*T. aestivum*	0.015	327	100	218	100	18	35.54	7.78/66.65	7.27/60.75
43	215398470	globulin 3	*T. aestivum*	0.047	251	100	184	100	14	23.5	7.78/66.65	7.75/60.16
46	215398470	globulin 3	*T. aestivum*	0.001	522	100	269	100	28	57.0	7.78/66.65	7.83/59.66
48	215398470	globulin 3	*T. aestivum*	0.025	704	100	466	100	28	49.5	7.78/66.65	8.04/59.21
394	215398470	globulin 3	*T. aestivum*	0.008	244	100	203	100	9	18.7	7.78/66.65	5.26/21.39
396	215398470	globulin 3	*T. aestivum*	0.001	116	100	78	99.994	10	17.5	7.78/66.65	5.86/21.13
280	122232330	Avenin-like b1	*T. aestivum*	0.037	246	100	209	100	5	22.1	8.08/33.79	8.14/35.36
302	110341801	globulin 1	*T. aestivum*	0.028	506	100	390	100	12	67.7	8.05/25.11	7.99/32.85
326	110341795	globulin 1	*T. aestivum*	0.075	631	100	433	100	18	80.9	8.57/25.10	8.67/30.27
380	215398472	globulin 3B	*T. aestivum*	0.0025	56	95.298	45	99.871	4	7.74	7.36/57.06	6.57/22.39
25	46981764	HMW glutenin subunit Dty10	*Ae. tauschii*	0.048	423	100	318	100	12	46.0	8.2/27.38	7.62/65.01
B15 d	171027826	triticin	*T. aestivum*	0.048	170	100	109	100	12	17.8	6.43/65.29	4.38/47.33
		**Carbon metabolism**										
116	525291	ATP synthase beta subunit	*T. aestivum*	0.003	623	100	467	100	19	52.8	5.56/59.33	5.04/52.00
247	357134729	glucose and ribitol dehydrogenase	*Brachypodium distachyon*	0.029	102	99.993	57	99.097	8	26.4	9.34/37.92	7.02/38.24
B10 d	253783729	glyceraldehyde-3-phosphate dehydrogenase	*T. aestivum*	0.010	341	100	213	100	15	40.9	6.67/36.62	7.44/40.96
		**Stress/defence/Det oxification**										
258	22001285	peroxidase 1	*T. aestivum*	0.028	408	100	228	100	19	45.53	8.14/39.26	9.18/37.12
443	326513238	Late embryogenesis abundant protein	*H. vulgare*	0.025	185	100	93	100	9	78.5	5.57/9.97	5.10/17.00
B21 d	54778511	0.19 dimeric alpha-amylase inhibitor	*T. aestivum*	0.018	176	100	90	100	8	79.83	6.49/13.76	5.84/17.00
		**unknown**										
342	326529599	predicted protein	*H. vulgare*	0.027	167	100	154	100	5	7.99	5.59/77.22	5.80/27.93
B16 d	326495978	predicted protein	*H. vulgare*	0.038	111	100	66	99.926	5	55.5	4.21/12.68	3.79/33.89

a Protein Score: statistical probability of true positive identification of the predicted protein calculated by MASCOT (score ≥ 42 against NCBInr).

b *TpI*/TMW (kDa): p*I* of predicted protein/molecular mass of predicted protein.

c *EpI*/EMW (kDa): p*I* of protein on the gel/molecular mass of protein on the gel.

d B1 was specially expressed in 3BS-8, and B15, B16, B21 were specially expressed in CS.

**Table 2:** The peptide sequences of differentially expressed proteins between CS and 3BS-8 identified by MS/MS.

Spot No.	Accession No.	Protein Name	±da	±ppm	Start Sequence	End Sequence	Peptide Sequence	Ion Score	Protein Score C.I.%
**storage proeins**									
25	gi|46981764	HMW glutenin subunit Dty10	0.0778	85	78	86	SVAVSQVAR	37	19.535
			0.0647	58	45	54	QVVDQQLAGR	71	99.966
			0.0563	43	34	44	ELQESSLEACR	72	99.975
			0.0288	20	227	239	AQQPATQLPTVCR	75	99.986
			−0.1294	−57	136	157	QGSYYPGQASPQQPGQGQQPGK	40	62.537
36	gi|215398470	globulin 3	−0.0166	−17	132	139	RPYVFGPR	44	83.599
			−0.0045	−4	154	163	ALRPFDEVSR	41	64.854
			0.0014	1	364	374	SFHALAQHDVR	72	99.974
			0.002	1	339	349	DTFNLLEQRPK	43	79.682
38	gi|215398470	globulin 3	−0.0068	−6	154	163	ALRPFDEVSR	37	21.378
			0.0028	2	364	374	SFHALAQHDVR	77	99.991
			0.01	7	339	349	DTFNLLEQRPK	50	95.444
43	gi|215398470	globulin 3	−0.0069	−6	154	163	ALRPFDEVSR	40	62.03
			−0.0053	−4	364	374	SFHALAQHDVR	61	99.703
			0.0043	3	339	349	DTFNLLEQRPK	64	99.857
46	gi|215398470	globulin 3	−0.019	−16	154	163	ALRPFDEVSR	41	68.692
			−0.0145	−11	364	374	SFHALAQHDVR	77	99.993
			−0.0069	−5	339	349	DTFNLLEQRPK	61	99.725
			0.0088	5	470	488	GSAFVVPPGHPVVEIASSR	45	89.119
			−0.0022	−1	446	464	GSGSESEEEQDQQRYETVR	44	86.858
48	gi|215398470	globulin 3	−0.0186	−16	154	163	ALRPFDEVSR	37	29.797
			−0.007	−5	364	374	SFHALAQHDVR	76	99.989
			0.0025	2	339	349	DTFNLLEQRPK	80	99.996
			0.0347	18	470	488	GSAFVVPPGHPVVEIASSR	101	100
			0.032	14	446	464	GSGSESEEEQDQQRYETVR	74	99.983
			0.06	25	542	562	AKDQQDEGFVAGPEQQQEHER	80	99.996
394	gi|215398470	globulin 3	0.1372	72	470	488	GSAFVVPPGHPVVEIASSR	77	99.994
			0.1484	67	446	464	GSGSESEEEQDQQRYETVR	60	99.733
			0.2031	84	542	562	AKDQQDEGFVAGPEQQQEHER	66	99.935
396	gi|215398470	globulin 3	0.0885	46	470	488	GSAFVVPPGHPVVEIASSR	61	99.742
280	gi|122232330	Avenin-like b1	0.069	55	203	212	QLSQIPEQFR	77	99.993
			0.0913	66	213	224	CQAIHNVAEAIR	92	100
			0.2202	74	225	248	QQQPQQQWQGMYQPQQPAQHESIR	39	60.794
302	gi|110341801	globulin 1	0.1034	87	47	56	QILEQQLTGR	74	99.988
			0.0934	67	35	46	GEVQEKPLLACR	69	99.958
			0.0948	63	99	111	DYEQSMPPLGEGR	42	81.431
			0.1016	56	57	74	AGEGAVGVPLFHAQWGAR	54	98.836
380	gi|215398472	globulin 3B	0.031	23	61	72	HGEGGREEEQGR	45	99.871
B15	gi|171027826	triticin	0.0056	3	61	77	SQAGLTEYFDEENEQFR	84	99.999
**Carbon metabolism**									
116	gi|525291	ATP synthase beta subunit	0.1179	85	249	262	AHGGFSVFAGVGER	108	100
			0.1168	83	148	161	VLNTGSPITVPVGR	72	99.983
			0.114	76	336	349	FTQANSEVSALLGR	79	99.996
			0.0671	33	413	431	QISELGIYPAVDPLDSTSR	53	98.601
			−0.0181	−8	192	211	EAPAFVEQATEQQILVTG	85	100
			−0.0049	−2	350	370	IPSAVGYQPTLATDLGGLGER	69	99.959
247	gi|357134729	glucose and ribitol dehydrogenase	−0.0207	−25	261	268	GAIVAFTR	31	0
			0.0351	29	97	109	VALVTGGDSGIGR	26	0
B10	gi|253783729	glyceraldehyde-3-phosphate dehydrogenase	0.0334	28	301	311	AGIALNDHFVK	46	89.006
			0.149	68	274	293	GIMGYVEEDLVSTDFVGDSR	100	100
			0.1816	82	274	293	GIMGYVEEDLVSTDFVGDSR	136	100
**Stress/defense/detoxification**									
258	gi|22001285	peroxidase 1	0.0365	37	62	71	DIGLAAGLLR	44	86.056
			0.0381	33	34	42	GLSFDFYRR	43	82.606
			0.0231	16	127	141	GAVVSCADILALAAR	73	99.982
443	gi|326513238	embryogenesis abundant protein	0.0187	14	30	41	SLEAQQNLAEGR	72	99.977
B21	gi|54778511	0.19 dimeric alpha-amylase inhibitor	0.0131	7	40	53	ECCQQLADISEWCR	54	98.884
unknown									
342	gi|326529599	predicted protein	0.0265	19	33	46	AGAAVGGQVVEKER	90	100
B16	gi|326495978	predicted protein	−0.058	−35	82	96	DIELVMTQAGVPRPK	35	9.731

**Table 3:** Specific primer sequences used for PCR amplification.

Primers	Sequences
gwm493	5wm493esATAACTAAAACCGCG-3 5TAACTAAAACCGCG-3 ers were s
gwm566	5wm566GTCTACCCATGGGATTTG-3s 5TCTACCCATGGGATTTG-3s were
